# Method Validation for Quantification of PFOS and PFOA in Human Plasma and a Pilot Study in Blood Donors from Thai Red Cross Society

**DOI:** 10.3390/toxics11121015

**Published:** 2023-12-13

**Authors:** Teerapong Lertassavakorn, Nanthanit Pholphana, Nuchanart Rangkadilok, Tawit Suriyo, Punthip Teeyapant, Jutamaad Satayavivad

**Affiliations:** 1 Laboratory of Pharmacology, Chulabhorn Research Institute, Bangkok 10210, Thailand; teerapong.ler@mahidol.ac.th (T.L.); nanthanit@cri.or.th (N.P.); nuchanart@cri.or.th (N.R.); tawit@cri.or.th (T.S.); 2 Center of Excellence on Environmental Health and Toxicology (EHT), OPS, MHESI, Bangkok 10400, Thailand; 3 Translational Research Unit, Chulabhorn Research Institute, Bangkok 10210, Thailand; punthip@cri.or.th; 4 Environmental Toxicology Program, Chulabhorn Graduate Institute (CGI), Bangkok 10210, Thailand

**Keywords:** PFOS, PFOA, LC–MS/MS, human plasma

## Abstract

Information regarding per- and polyfluorinated substances concentrations in biological samples from the Thai population was still lacking. A sensitive bioanalytical method was developed and validated for the quantification of perfluorooctane sulfonic acid (PFOS) and perfluorooctanoic acid (PFOA) levels in human plasma. Simple protein precipitation and LC–MS/MS techniques were used with stable isotope internal standards of ^13^C_8_–PFOS and ^13^C_8_–PFOA. The validated method followed the ICH bioanalytical validation guideline, and the results showed good accuracy, precision, and reproducibility. The validated analytical method was then applied to determine PFOS and PFOA concentrations in 50 human plasma samples from the National Blood Center, Thai Red Cross Society. The concentrations were found to be in ranges of <0.91–6.27 ng/mL for PFOS and <0.49–2.72 ng/mL for PFOA. PFOS was also measured separately for its isomers, and the geometric means of the linear isomer (L–PFOS) and branched isomer (br–PFOS) in plasma samples were at 1.85 and 0.41 ng/mL, respectively. Both PFOS and PFOA concentrations were lower in comparison to previous reports from other countries. The present study showed the application of our reliable method to determine PFOS and PFOA in biological samples in order to monitor the human exposure of both chemicals in Thailand.

## 1. Introduction

Per- and polyfluoroalkyl substances (PFAS) are fluorinated synthetic chemicals applied in various consumer products, including stain-resistant coatings, pesticides, and paper-based food packaging [1,2,3]. The two most widely studied PFAS, perfluorooctane sulfonic acid (PFOS) and perfluorooctanoic acid (PFOA), are considered as the persistent and bioaccumulative compounds that contaminate much in the environment and organisms [4,5]. Both compounds are proved to be persistent in the human body, as the estimated mean half-life was 3.4 years for PFOS and 2.7 years for PFOA [6]. Significant associations were observed in several studies between human exposure to PFOS and PFOA and clinical outcomes, for example, thyroid hormone disruption, low birth weight, dysregulation of lipid and glucose homeostasis, and immunotoxicity [7,8,9,10].

Previous studies in Thailand have determined PFOS and PFOA concentrations in water [11,12], consumer goods [13], and seafood [14]. Even though there are available data on the contamination of PFAS in the environment and food, there is still no report on PFAS exposure in the Thai population yet. Several countries have monitored the concentrations of PFOS and PFOA in human biological samples, for instance, the National Report on Human Exposure to Environmental Chemicals (NRHEEC) by the Centers for Disease Control and Prevention of the United States or the joint collaboration of European countries in the European Human Biomonitoring Initiative (HBM4EU). These biomonitoring assessments provided information about PFAS exposure levels and potential sources in a study population from different regions [15]. The data from the human body also served as a basis for ascertaining the risk between PFOS and PFOA exposure and health outcomes. In addition, the PFAS isomeric composition should be distinctly determined, as previous studies strongly suggested that the PFAS isomers exhibited different toxicities to human health [16,17,18].

Therefore, the objectives of the present study were to develop and validate an extraction method and a sensitive LC–MS/MS technique for the determination of PFOS (linear, branch, and total forms) and PFOA in human plasma. The validation protocol followed the ICH M10 on bioanalytical method validation [19]. Additionally, a preliminary study was also conducted by implementing this validated method to analyze PFOS and PFOA concentrations in the plasma samples of blood donors obtained from the Thai Red Cross Society.

## 2. Materials and Methods

### 2.1. Chemicals and Reagents

PFOA (purity > 98%) and br–PFOSK (purity ≥ 98%, 78.8% of linear isomer and 21.2% branch isomer compositions) were purchased from Wellington Laboratories (Guelph, ON, Canada). Isotopically labeled internal standards [^13^C_8_–PFOS (purity 99%) and ^13^C_8_–PFOA (purity 99%)] were purchased from Cambridge Isotope Laboratories (Andover, MA, USA). HPLC-grade methanol (MeOH), ammonium acetate, glacial acetic acid, and EmbryoMax® fetal bovine serum were purchased from Merck (Kenilworth, NJ, USA). Acetonitrile was purchased from Honeywell (Charlotte, NC, USA). Ultrapure water (Type I) was obtained from the Milli-Q system (Millipore, Bedford, MA, USA). 

### 2.2. Preparation of Stock and Working Solutions

A total of 0.1 mL of PFOS and PFOA (50 μg/mL in methanol, 0.05 mL each) was diluted with 0.4 mL of acetonitrile to obtain a mixed stock solution of 5 µg/mL. This standard solution was used to prepare working standards covering the concentration ranges from 20 to 900 ng/mL for PFOS and 10 to 600 ng/mL for PFOA. It is worth noting that PFOS consists of two isomers, including the linear isomer (L–PFOS; 78.8% isomer composition) and the branched isomer (br–PFOS; 21.2% isomer composition). Both L–PFOS and br–PFOS were prepared as individual substances with their own distinct concentration ranges. The isotopically labeled internal standards (IS), ^13^C_8_–PFOS and ^13^C_8_–PFOA (50 μg/mL in methanol), were also diluted in acetonitrile to achieve the mixed stock solution of 5 µg/mL. The IS solution was further diluted in acetonitrile to obtain a final concentration of 5 ng/mL. For QCs standard preparation, 0.05 mL of each standard PFOS and PFOA (50 μg/mL in methanol) was diluted with 0.45 mL of acetonitrile to obtain the individual stock solution of PFOS and PFOA at 5 μg/mL. These QCs standard solutions were used to prepare QCs working standards (LLOQ, LQC, MQC, and HQC) covering the concentration ranges from 20 to 700 ng/mL for PFOS and 10 to 500 ng/mL for PFOA. All stock and working solutions were kept at 2–8 °C in laboratory refrigerator.

### 2.3. Preparation of Calibration Standards and Quality Controls (QC)

The calibration standards were prepared by spiking PFOS and PFOA working standards into the fetal bovine serum (FBS) matrix to obtain the final concentrations in a range of 0.91–41.01 ng/mL for PFOS (0.72–32.32 ng/mL for L–PFOS and 0.19–8.69 ng/mL for br–PFOS) and 0.49–29.40 ng/mL for PFOA. Quality controls (QC) samples were also prepared in the FBS matrix at four concentration levels, including lower limit of quantification (LLOQ; L–PFOS = 0.72 ng/mL, br–PFOS = 0.19 ng/mL, and PFOA = 0.49 ng/mL), low QC (LQC; L–PFOS = 1.80 ng/mL, br–PFOS = 0.48 ng/mL, and PFOA = 0.98 ng/mL), medium QC (L–PFOS = 10.77 ng/mL, br–PFOS = 2.9 ng/mL, and PFOA = 9.8 ng/mL), and high QC (HQC; L–PFOS = 25.14 ng/mL, br–PFOS = 6.76 ng/mL, and PFOA = 24.5 ng/mL). In addition, the MQC and HQC were also prepared in human plasma matrix.

### 2.4. Sample Preparation and Extraction

Human plasma samples used for method validation in this bioanalytical method development and validation were acquired from the National Blood Center, Thai Red Cross Society (Protocol number: 2/2021; approved by Research Ethics Committee, National Blood Center, Thai Red Cross Society). An extraction method using acetonitrile protein precipitation was modified from Olsen et al. [20]. The collected frozen plasma samples were thawed at room temperature. An aliquot of 0.1 mL human plasma was put into a 1.5 mL microcentrifuge tube. Then 0.3 mL of acetonitrile containing mixed-labeled IS (^13^C_8_–PFOS and ^13^C_8_–PFOA at 5 ng/mL) was added into the samples, vortex mixed for 1 min, and centrifuged at 11,000× *g* for 10 min (4 °C). Lastly, the supernatant of 0.1 mL was transferred into an amber polypropylene autosampler vial for LC–MS/MS analysis. All plasma samples and their extracts were kept out of contact with any PFAS-containing materials, as well as glass containers, to prevent PFAS losses from sorption.

### 2.5. Instrumentation

The LC–MS/MS analysis of human plasma samples was performed using an Agilent 1290 Infinity UHPLC coupled with an Agilent 6490 Triple Quadrupole MS/MS (Agilent, Santa Clara, CA, USA). Chromatographic separation was conducted using an Agilent ZORBAX Eclipse Plus C18 (2.1 × 50 mm, 1.8 μm) column connected to an UHPLC Guard (Poroshell 120 EC–C18). For the mobile phase, 5 mM ammonium acetate with 0.01% acetic acid (solvent A) and 50:50 *v*/*v* of MeOH and acetonitrile (solvent B) were implemented. A gradient system with a pump flow rate of 0.2 mL/min was operated in the following steps: 0–1.00 min (40% B), 1.00–1.01 min (40–60% B), 1.01–5.50 min (60–68% B), 5.50–5.60 min (68–40% B), and maintained at 40% B for 2.40 min (8 min of total run time). The injection volume was 5 µL. The temperatures of the column and autosampler tray were maintained at 40 °C and 4 °C, respectively. Mass spectrometry was operated in the negative electrospray ionization (ESI) and multiple reaction monitoring (MRM) modes. The gas temperature was maintained at 170 °C with a flow rate of 11 L/min. A nebulizer pressure of 45 psi was applied. Capillary and nozzle voltages were set at −3000 V and −500 V, respectively. The MRM transition and collision energy for each substance are listed in Table S1.

### 2.6. Bioanalytical Method Validation

The validation of bioanalytical method was conducted according to the International Council for Harmonization of Technical Requirements for Pharmaceuticals for Human Use or ICH guideline [19]. The validation parameters and their acceptance criteria are described as follows.

#### 2.6.1. Selectivity and Specificity 

The selectivity and specificity were assessed by determining the characteristics of 10 blank human plasma samples acquired from the National Blood Center, Thai Red Cross Society. These blank samples should be free of interference at the retention times of PFOS and PFOA, as well as the IS. The detected responses of analytes and IS in blank samples should not be more than 20% at the LLOQ level and 5% of the IS response at the LLOQ level.

#### 2.6.2. Linearity 

To derive linearity, the calibration in each run consisted of a blank sample (no analytes and IS), a zero sample (a blank sample with IS), seven non-zero samples for PFOS (0.72–32.32 ng/mL for L–PFOS and 0.19–8.69 ng/mL for br–PFOS), and seven non-zero samples for PFOA (0.48–29.40 ng/mL). The accuracy of the back-calculated concentrations of each non-zero sample should be within ±15% except at LLOQ (within ±20%), and at least 75% of the non-zero samples should fulfill the above criteria.

#### 2.6.3. Accuracy and Precision

Within run (in each run) and between run (in a different run) accuracy and precision tests were evaluated in 3 independent analytical runs. Four QCs prepared in the surrogate matrix of FBS (LLOQ, LQC, MQC, and HQC; 7 replicates per QC level) were analyzed to determine the method’s accuracy and precision. In addition, two QCs were also prepared in human plasma at medium and high concentration levels (MQC and HQC, 7 replicates per QC level) in order to simulate the real plasma sample. The %recovery should be within ±15% of the nominal concentration, except at LLOQ (within ±20%) to be considered acceptable accuracy. For precision, the coefficient of variation (%CV) should not exceed ±15%, except at LLOQ, where %CV was acceptable up to ±20%.

The standard reference material, SRM 1957–organic contaminants in non-fortified human serum (freeze-dried) from National Institute of Standards and Technology (NIST), was also analyzed in this study as one of the QCs for accurate measurement of PFOS and PFOA in human plasma samples. The reference mass fraction values were 21.1 ± 1.3 µg/kg for PFOS (a total of L–PFOS and br–PFOS) and 5.00 ± 0.44 µg/kg for PFOA.

#### 2.6.4. Carry-Over

An injection of the upper limit of quantification (ULOQ; 32.32 ng/mL for L–PFOS, 8.69 ng/mL for br–PFOS, and 29.40 ng/mL for PFOA) prepared in the FBS matrix followed by the blank FBS sample was analyzed to evaluate carry-over effect. The response shown in the subsequent blank sample should not be greater than 20% at the LLOQ level and 5% for the IS.

#### 2.6.5. Matrix Effect

According to the ICH bioanalytical guideline [19], if the surrogate matrix approach is used, the impact of the matrix effect between QCs in the surrogate and original matrix should be investigated to determine whether they are similar or not. The human plasma from the selectivity test was prepared as MQC and HQC and used to determine the matrix effect against the FBS calibration curve. The accuracy (%recovery) should be within ±15% of the nominal concentration, and the precision (%CV) should not be greater than 15%.

#### 2.6.6. Recovery

Recovery or extraction efficiency was evaluated by comparing the analyte response in the matrix spiked with analyte before and after extraction. The QC for FBS matrix included LQC, MQC, and HQC (5 replicates per level), and for human plasma matrix, MQC and HQC were prepared with 5 replicates per level. The recovery of the analyte and IS should be consistent throughout the analytical run.

#### 2.6.7. Stability

The stability of PFOS and of PFOA were assessed under several storage conditions, including stock and working solution stability, bench-top (short-term) stability, processed sample stability, freeze–thaw stability, and long-term stability. LQC and HQC (5 replicates each per storage condition) were analyzed for their accuracy, which should be within ±15% of the QC nominal concentration, to confirm that the storage conditions did not affect the analyte’s concentration. The stability tests were examined against freshly prepared calibration standards and QC.

- Stock and working solutions stability were determined by storing the standards in a laboratory refrigerator at 2–8 °C up to 6 days and analyzing the accuracy.
- Bench-top stability was assessed after thawing the QC samples at room temperature for 6 h before the analysis.
- Processed sample stability was measured by investigating the extracted QC samples kept in an autosampler at 4 °C for 24 h.
- Freeze–thaw stability required the QC samples to be stored at −80 °C in the freezer for at least 12 h and thawed at room temperature for at least 1 h. This freeze–thaw cycle was carried out up to three cycles before conducting the analysis.
- Long-term stability: the QC samples were stored at −80 °C in the freezer for 1 month before analysis.

#### 2.6.8. Dilution Integrity

In order to adequately measure concentrations above the ULOQ, a QC sample with a concentration higher than the ULOQ and its dilution should be prepared and tested for accuracy and precision. The human plasma matrix was spiked to achieve 64.64, 17.39, and 58.8 ng/mL for L–PFOS, br–PFOS, and PFOA, respectively, before undergoing dilution with the same human plasma. The dilution procedure was performed with two dilution factors, including 1:2 and 1:4 ratios. Both the %recovery and %CV should be within ±15%.

### 2.7. Application to Human Plasma Samples

A preliminary study was conducted in 50 human plasma samples from healthy Thai blood donors (age 17–70 years). The samples were acquired from the National Blood Center, Thai Red Cross Society (Protocol number: 20/2021) with ethical approval from the Research Ethics Committee, National Blood Center, Thai Red Cross Society. Detection frequencies were calculated as a percentage (%) by dividing the number of plasma samples detected with PFOS and PFOA concentrations above the LLOQ by the number of total plasma samples.

## 3. Results

### 3.1. Validation of Bioanalytical Method for Determination of PFOS and PFOA in Human Plasma

#### 3.1.1. Selectivity and Specificity

For the selectivity and specificity tests, the endogenous levels at the retention time of both PFOS and PFOA in 10 analyzed plasma samples showed responses greater than 20% of their respective LLOQ levels (Figures S1 and S2). Because of the interferences, the surrogate matrix approach was applied in order to mimic the human plasma matrix. Therefore, fetal bovine serum (FBS) was selected as a substitute for the human blank samples throughout the study. The surrogate matrix had an endogenous response less than 20% of the LLOQ (7.05% for L–PFOS, 16.42% for br–PFOS, and 8.52% for PFOA), and not more than 5% of the IS response of the LLOQ (0.04% for PFOS and 0.003% for PFOA).

#### 3.1.2. Linearity 

Linear regression analysis with the weighting of 1/x of the PFOS and PFOA calibration standards showed good correlation coefficients (r ≥ 0.997) throughout the validation run. The back-calculated concentrations of the calibration standards were in the range of 101.57–116.12%, which were within 20% of their respective LLOQ levels. The rest of the non-zero samples also had back-calculated concentrations within 15% of the nominal concentration (90.99–105.54%).

#### 3.1.3. Accuracy and Precision

The accuracy (%recovery) of the analyzed QC in FBS for PFOS and PFOA within each run (intra-day) and in between runs (inter-day) varied between 105.42% and 110.68% and 110.07% and 114.31%, respectively, for LLOQ. As for the other QC (LQC, MQC, and HQC), the accuracy of intra-day and inter-day assays were in the ranges of 95.17–99.12% and 97.48–106.21%, respectively (Table 1). The precision (%CV) for each and different runs were all less than 10% (2.06–6.86%), which complied with the bioanalytical guideline [19]. The results of the accuracy and precision parameters in the human plasma matrix (MQC and HQC) were in the ranges of 92.11–102.69% and 1.37–5.21%, respectively, which were also in the acceptance criteria (Table 1). Moreover, the result of SRM–1957 QC showed good accuracy, with a mean %recovery of 101.93% for PFOS and 104.76% for PFOA.

#### 3.1.4. Carry-Over

After analyzing the blank sample after the injection of the ULOQ, the responses of each analyte were not greater than 20% of LLOQ responses as follows: 12.74% for total PFOS, 5.22% for L–PFOS, 18.25% for br–PFOS, and 15.30% for PFOA. For internal standards, the responses shown in the blank sample were all less than 5%, indicating no carry–over.

#### 3.1.5. Matrix Effect and Extraction Efficiency

The validation assay proved that the human plasma had no matrix effect, as the accuracy was found to be at 99.87–105.48% for PFOS, and 99.21–106.05% for PFOA (Table 2). Even though the ICH guideline did not indicate a specific range of accuracy to comply with when comparing pre- and post-extraction efficiency [19], the %recovery should be consistent throughout the validation run. The mean recovery for both PFOS and PFOA compounds was within ±15%, with the exception of HQC for br–PFOS with a mean recovery of 115.25% (Table 2).

#### 3.1.6. Stability

The results of PFOS and PFOA integrity in FBS QCs that were subjected to storage conditions with varying times and temperatures are listed in Table 3. The accuracy and precision of the analytes were all in compliance with the ICH bioanalytical guideline [19].

#### 3.1.7. Dilution Integrity

The integrity of PFOS and PFOA in human plasma in the dilution process remained consistent from the validation run. The means of %recovery and %CV of the 1:2 dilution factor for L–PFOS, br–PFOS, total PFOS, and PFOA were 100.09%, 101.87%, 100.29%, and 105.37%, respectively, and 2.13%, 2.68%, 2.19%, and 1.20%, respectively. As for the 1:4 dilution factor, the mean accuracies were in the range of 99.05–101.93%, and the mean precisions were in the range of 1.84–2.04%.

### 3.2. A Pilot Study in Thai Blood Donors Plasma Samples

The validated method from this study was utilized in human plasma samples from healthy Thai blood donors as a means to determine PFOS and PFOA concentrations. A total of 50 plasma samples were analyzed, and the levels of total PFOS and PFOA were in the ranges of <0.91–6.27 ng/mL (median = 2.70 ng/mL) and <0.49–2.72 ng/mL (median = 0.79 ng/mL), respectively (Table 4 and Table S2). More than 85% of the blood donors’ plasma samples were detected with either PFOS or PFOA. For the isomers of PFOS, L–PFOS and br–PFOS concentrations ranged from <0.72 to 5.12 ng/mL and from <0.19 to 1.31 ng/mL, respectively. Interestingly, the average isomer abundance of linear isomers to total levels of PFOS in the sample group was 81.51%. It should be noted that the plasma samples with PFOS and PFOA below the LLOQ levels were substituted with LLOQ/2 before analyzing for any statistical parameter according to the USEPA guidance [21].

## 4. Discussion

The results of the developed method for determination of PFOS and PFOA concentrations in human plasma were satisfactory, as all key features from the validation assay, including selectivity/specificity, linearity, accuracy and precision, matrix effect, stability, extraction efficiency, carry-over, and dilution integrity, were in accordance with the international bioanalytical validation guidelines [19]. Moreover, the modified extraction procedure also used a small volume of plasma samples (0.1 mL), simple protein precipitation with acetonitrile, and a few chemicals (no addition of phosphoric acid and water) compared to the previous method [20], without affecting the integrity of analytes. Even though other extraction procedures, including the ion-pair preparation method, solid-phase extraction (SPE) [22], or a combination of protein precipitation and online-SPE [23], could also be used to quantify concentrations of PFOS and PFOA from human serum or plasma samples, our extraction method was notably easy and simple to conduct with low LLOQ value. Therefore, our developed method was reliable for the determination of PFOS and PFOA concentrations in human plasma. 

**Figure 1:**
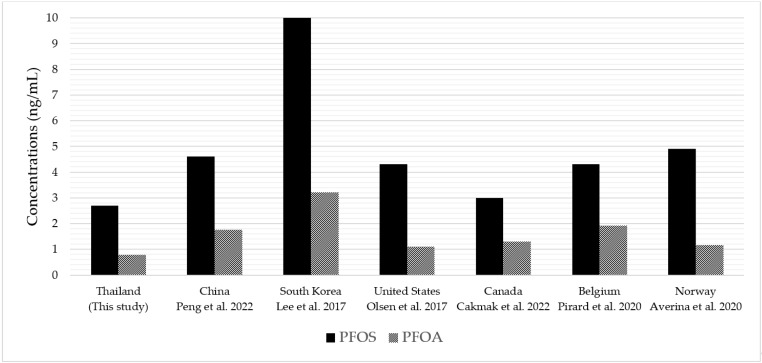
Comparison of average PFOS and PFOA concentrations in human serum/plasma from the general population in different countries and Thailand in the present study [20,24,27,29,30,31].

The present study is the first to report the PFOS and PFOA concentrations in plasma samples of the Thai population, specifically of blood donors from the Thai Red Cross Society. The results indicate that both PFOS and PFOA concentrations found in these plasma samples were lower when compared to general populations in other countries (Figure 1). Most of the blood samples from the general population in various countries had higher concentrations of PFOS than PFOA. In certain developed countries, high concentrations of PFOS and PFOA were found in human blood samples, up to 29.40 and 13.30 ng/mL, respectively, in the United States [20], and 214.26 and 18.69 ng/mL, respectively, in South Korea [24]. Elevated concentrations of PFOS and PFOA were also observed in the European population. A study on the middle-aged general population of Uppsala, Sweden, reported average concentrations of 9.10 ng/mL for PFOS and 2.40 ng/mL for PFOA in serum samples [25]. In Italy, geometric means of PFOS and PFOA concentrations in serum were 6.20 and 1.60 ng/mL, respectively, in the general population of the Veneto region [26]. Both compounds were also extensively reported in plasma or serum from residences in China, especially the industrialized cities, for instance, Shenyang (up to 64.78 ng/mL for PFOS and 135.14 ng/mL for PFOA) and Guangzhou (up to 352.24 ng/mL for PFOS and 10.06 ng/mL for PFOA) [16,27], as the country is now the world’s largest PFAS producer [28].

The abundance of PFOS isomers was also determined in the present study, as previous research found that the compound’s linear and branched isomers possessed unique adverse health outcomes upon exposure [32]. Recently, a joint research collaboration in China, namely, “the Isomers of C8 Health Project”, detailed the specific effects of PFOS exposure, including liver toxicity [17], obesity in women [18], and kidney dysfunction [16]. The present study found the relative amount of L–PFOS to total concentrations of PFOS in the blood donor’s subgroup in a range of 69.82–90.08% (Table 5). Participants in a Canadian prospective birth cohort study had an average PFOS linear isomer composition of 69.00% [33]. Meanwhile, nearly 96.71% of the L–PFOS isomer was found in the serum of middle-aged Taiwanese adults [34]. Interestingly, the average L–PFOS isomer proportion found in the serum samples of aviation firefighters from Australia with historical exposure to aqueous-film-forming foam was only 35% [35]. Consequently, differences in the PFOS isomer composition should be considered in the interpretation of any PFAS-related results, as the health outcomes could vary greatly for certain study populations. 

However, this pilot study had several limitations. Firstly, the sample size was too small to represent the background concentrations of PFOS and PFOA in the general Thai population. Secondly, the plasma samples acquired from the Thai Red Cross Society had no descriptive information, for instance, age, gender, or occupation. Therefore, an accurate assessment linking the PFAS concentrations to the personal data of any given plasma samples could not be established.

## 5. Conclusions

A simple extraction method with the use of a small sample volume and a sensitive LC–MS/MS technique for the quantification of PFOS (linear, branched, and total forms) and PFOA in human plasma were validated by following all essential parameters listed in the ICH bioanalytical guidelines. In addition, the developed method was utilized in determining the concentrations of both compounds in the plasma samples of Thai blood donors. Our preliminary findings found that PFOS and PFOA concentrations in human plasma were relatively low when compared to the previous reports from the general population in other countries. Further study is needed to confirm the results of this finding due to the lack of sample size and descriptive information on each biological sample. Moreover, other classes of PFAS should be included and quantified in the next study in order to achieve a thorough overview of the PFAS contamination situation in Thai population.

## Figures and Tables

**Table 1:** Accuracy and precision of within run (intra-day, *n* = 7 per QC) and between run (inter-day, *n* = 21 per QC) of PFOS and PFOA in fetal bovine serum (FBS) and human plasma matrices.

Compounds	QC Level (ng/mL)	Intra-Day (*n* = 7)		Inter-Day (*n* = 21)	
		%Recovery	%CV	%Recovery	%CV
Fetal bovine serum matrix					
L–PFOS	LLOQ (0.72)	107.78	4.91	112.53	4.74
	LQC (1.80)	95.74	4.55	99.65	5.49
	MQC (10.77)	95.36	2.94	97.78	5.16
	HQC (25.14)	98.84	2.74	102.23	4.87
br–PFOS	LLOQ (0.19)	105.42	4.33	110.07	5.02
	LQC (0.48)	95.53	5.24	101.44	6.86
	MQC (2.90)	96.13	2.81	99.08	4.89
	HQC (6.76)	98.34	2.17	101.42	4.21
Total PFOS	LLOQ (0.91)	109.08	3.68	114.31	4.09
	LQC (2.28)	95.81	4.36	100.70	5.75
	MQC (13.67)	95.38	2.59	97.48	4.13
	HQC (31.90)	98.72	2.39	101.89	4.52
PFOA	LLOQ (0.49)	110.68	3.78	110.88	3.22
	LQC (0.98)	98.07	3.68	100.71	4.55
	MQC (9.80)	95.17	2.06	98.23	6.00
	HQC (24.50)	99.12	3.05	106.21	6.09
Human plasma matrix					
L–PFOS	MQC (10.77)	96.07	2.44	98.02	4.26
	HQC (25.14)	97.52	1.49	101.19	3.84
br–PFOS	MQC (2.90)	95.15	3.18	95.77	4.20
	HQC (6.76)	96.29	2.05	99.97	3.44
Total PFOS	MQC (13.67)	95.27	1.37	97.06	4.03
	HQC (31.90)	97.35	1.62	101.00	3.67
PFOA	MQC (9.80)	92.11	1.72	94.30	4.15
	HQC (24.50)	95.98	3.67	102.69	5.21

**Table 2:** Average accuracy (%recovery) of PFOS and PFOA acquired from matrix effect and extraction efficiency.

Compounds	QC Levels	Recovery (*n* = 5/QC)		Matrix Effect (*n* = 5/QC)
		FBS	Human Plasma	
L–PFOS	LQC (1.80)	102.21		
	MQC (10.77)	99.16	105.73	103.11
	HQC (25.14)	113.94	106.19	105.48
br–PFOS	LQC (0.48)	100.73		
	MQC (2.90)	101.48	106.12	99.87
	HQC (6.76)	115.25	106.07	102.78
Total PFOS	LQC (2.28)	101.78		
	MQC (13.67)	99.58	106.58	101.95
	HQC (31.90)	114.41	106.19	104.79
PFOA	LQC (0.98)	106.25		
	MQC (9.80)	107.04	100.05	99.21
	HQC (24.50)	111.14	102.66	106.05

**Table 3:** Accuracy and precision of PFOS and PFOA in biological matrices kept in distinct storage conditions.

Compounds	QC Levels	Storage Conditions for Stability Tests (*n* = 5/QC)							
		Bench-Top (6 h, 25 °C)		Processed Samples (24 h, 4 °C)		Freeze–Thaw (3 Cycles, −80 °C)		Long-Term (1 Month, −80 °C)	
		%Recovery	%CV	%Recovery	%CV	%Recovery	%CV	%Recovery	%CV
L–PFOS	LQC	104.75	2.00	110.49	2.84	100.21	1.33	101.19	1.66
	HQC	96.31	1.87	110.54	1.66	105.48	2.82	96.29	2.67
br–PFOS	LQC	105.60	4.22	111.50	4.71	105.42	4.01	105.98	1.32
	HQC	95.77	2.17	109.08	2.57	102.51	2.06	94.13	1.58
Total PFOS	LQC	105.70	1.54	112.42	1.08	101.03	2.32	101.73	1.65
	HQC	96.75	1.57	110.02	2.12	105.76	1.48	97.69	2.84
PFOA	LQC	104.14	4.00	113.45	2.39	114.39	3.25	106.29	2.05
	HQC	92.66	1.56	102.07	2.33	102.27	13.78	93.16	2.12

**Table 4:** PFOS and PFOA concentrations in plasma of Thai blood donors obtained from the Thai Red Blood Cross Society (*n* = 50).

Parameters	Analytes			
	L–PFOS	br–PFOS	Total PFOS	PFOA
Detection frequencies (%)	94	86	92	86
Minimum (ng/mL)	<0.72	<0.19	<0.91	<0.49
50th percentile (ng/mL)	2.21	0.49	2.62	0.79
Maximum (ng/mL)	5.12	1.31	6.27	2.72
Geometric mean (ng/mL)	1.85	0.41	2.26	0.83

**Table 5:** Composition of linear PFOS isomer to total PFOS in human serum/plasma from several studies.

Countries	Sampling Years	Population Group (N)	Average L-PFOS Isomers (%)	References
Australia	2016–2017	Australian volunteers (2400)	77.76	[36]
	2018–2019	Aviation firefighters (120)	35.00	[35]
Canada	2009–2012	Womens prior to 18 weeks of gestation (494)	69.00	[33]
China	2013	Cord blood samples of volunteers in Guangzhou (321)	75.16	[37]
	2015–2016	General residents of Shenyang city (1612)	49.23	[16]
Sweden	2014–2017	Pooled serum from first-time mothers in Uppsala (120)	71.45	[38]
Taiwan	2009–2011	Taiwanese participants age 22–63 years (597)	96.71	[34]
United States	2015–2016	U.S. population from NHANES survery (1993)	67.80	[39]
	2017–2018	U.S. population from NHANES survery (1929)	69.18	
Thailand	2021	Red Cross blood donors (50)	81.51	This study

## Data Availability

Data are contained within the article and supplementary materials.

## Supplementary Materials

The following supporting information can be downloaded at https://www.mdpi.com/article/10.3390/toxics11121015/s1, Table S1: Mass spectrometry parameters for PFOS and PFOA, and IS. Table S2: PFOS and PFOA concentrations in Thai blood donors’ plasma samples acquired from the Thai Red Blood Cross Society (*n* = 60). Figure S1—Extracted ion chromatogram of L–PFOS and br–PFOS in each biological matrix from selectivity study: (A–J) A total of 10 Thai blood donors’ blank plasma samples; (K) FBS blank sample without endogenous interference; (L) FBS blank sample spiked at LLOQ level (L–PFOS = 0.72 ng/mL, br–PFOS = 0.19 ng/mL). Figure S2—Extracted ion chromatogram of PFOA in each biological matrix from selectivity study: (A–J) A total of 10 Thai blood donors’ blank plasma samples; (K) FBS blank sample without endogenous interference; (L) FBS blank sample spiked at LLOQ level (PFOA = 0.49 ng/mL). Figure S3: Extracted ion chromatograms of PFOA and PFOS standards and their isotopically labeled internal standards (^13^C_8_–PFOA and ^13^C_8_–PFOS).
