# Supplementary material for: Method Validation for Quantification of PFOS and PFOA in Human Plasma and a Pilot Study in Blood Donors from Thai Red Cross Society
Source: Toxics. 2023 Dec 13;11(12):1015. doi: 10.3390/toxics11121015 (PMC10747079; doi:10.3390/toxics11121015)
Supplement: Supplementary file 1 [file toxics-11-01015-s001.zip › toxics-2713021-supplementary.pdf]

Supplementary Information

Method Validation for Quantification of PFOS and PFOA in Human Plasma and A Pilot Study in Blood Donors from Thai Red Cross Society

Table S1 Mass spectrometry specific parameters for PFOS and PFOA, and IS

Analyte	MRM transition (m/z)	Collision energy (volt)
PFOS	498.9 → 80.0*	–55
	498.9 → 99.0	–50
	498.9 → 130.0	–50
PFOA	412.9 → 368.9*	–6
	412.9 → 169.0	–18
	412.9 → 219.0	–15
$^{13}C_8$-PFOS**	506.9 → 80.0*	–55
	506.9 → 98.8	–55
$^{13}C_8$-PFOA	420.8 → 375.9*	–5
	420.8 → 172.1	–10

Note: *Select precursor-to-product ion used for quantification
**Both L–PFOS and br–PFOS used the same isotopic-labelled internal standard

Table S2 PFOS and PFOA concentrations in Thai blood donor's plasma samples acquired from the Thai Red Cross Society (n=60)

**Plasma samples**	**Analyte concentrations (ng/mL)**			
	**PFOA**	**L-PFOS**	**br-PFOS**	**Total PFOS**
**SL–1**	0.49	2.71	0.57	3.28
**SL–2**	0.79	2.10	0.68	2.78
**SL–3**	1.33	3.54	1.07	4.60
**SL–4**	0.74	1.96	0.59	2.55
**SL–5**	0.86	1.19	0.47	1.66
**SL–6**	0.80	1.08	0.50	1.58
**SL–7**	2.09	2.29	0.52	2.81
**SL–8**	0.71	1.93	0.51	2.44
**SL–9**	1.46	1.76	0.77	2.53
**SL–10**	1.44	3.09	1.13	4.22
**PS-1**	1.07	4.51	0.93	5.44
**PS-2**	1.19	2.23	0.53	2.76
**PS-3**	1.82	4.69	1.08	5.77
**PS-4**	0.77	2.01	0.46	2.48
**PS-5**	1.19	2.56	0.58	3.13
**PS-6**	1.17	2.83	1.22	4.05
**PS-7**	2.27	3.32	0.88	4.20
**PS-8**	0.78	1.14	0.34	1.48
**PS-9**	0.78	1.87	0.31	2.18
**PS-10**	0.59	0.95	0.23	1.18
**PS-11**	2.34	2.45	0.57	3.01
**PS-12**	0.54	1.19	<0.19	1.38
**PS-13**	0.64	1.59	0.24	1.83
**PS-14**	0.68	1.24	0.28	1.52
**PS-15**	1.34	2.33	0.45	2.78
**PS-16**	0.51	2.19	0.44	2.63
**PS-17**	1.06	2.23	0.40	2.63
**PS-18**	0.66	3.03	0.76	3.79
**PS-19**	<0.49	0.74	0.20	0.94
**PS-20**	0.76	3.20	0.52	3.72

Plasma samples	Analyte concentrations (ng/mL)			
	PFOA	L-PFOS	br-PFOS	Total PFOS
**PS-21**	1.80	2.92	0.84	3.76
**PS-22**	1.66	3.27	1.12	4.39
**PS-23**	0.79	1.26	0.32	1.58
**PS-24**	0.76	5.20	0.96	6.16
**PS-25**	0.62	1.21	0.33	1.54
**PS-26**	0.77	4.27	0.71	4.98
**PS-27**	<0.49	<0.72	<0.19	<0.91
**PS-28**	0.78	2.78	0.53	3.31
**PS-29**	1.12	2.01	0.50	2.51
**PS-30**	1.26	2.28	0.58	2.85
**PS-31**	<0.49	1.09	<0.19	1.23
**PS-32**	0.77	0.72	0.21	0.93
**PS-33**	1.20	1.71	0.58	2.28
**PS-34**	<0.49	<0.72	<0.19	<0.91
**PS-35**	1.22	2.86	0.54	3.40
**PS-36**	0.79	1.43	0.31	1.74
**PS-37**	0.66	2.95	0.67	3.62
**PS-38**	1.88	2.86	0.82	3.68
**PS-39**	1.28	3.24	0.82	4.06
**PS-40**	<0.49	<0.72	<0.19	<0.91
**PS-41**	<0.49	0.76	<0.19	<0.91
**PS-42**	1.19	1.18	0.41	1.59
**PS-43**	0.84	2.30	0.31	2.61
**PS-44**	1.00	1.90	0.43	2.33
**PS-45**	0.62	2.79	0.60	3.39
**PS-46**	1.32	3.26	0.88	4.14
**PS-47**	<0.49	0.79	<0.19	0.92
**PS-48**	1.32	1.78	0.49	2.26
**PS-49**	0.94	1.63	0.52	2.15
**PS-50**	2.72	4.96	1.31	6.27

Note: SL=Plasma samples from selectivity tests; PS=Plasma samples from preliminary study
LLOQ (ng/mL): L–PFOS (0.72), br–PFOS (0.19), Total PFOS (0.91), PFOA (0.49)

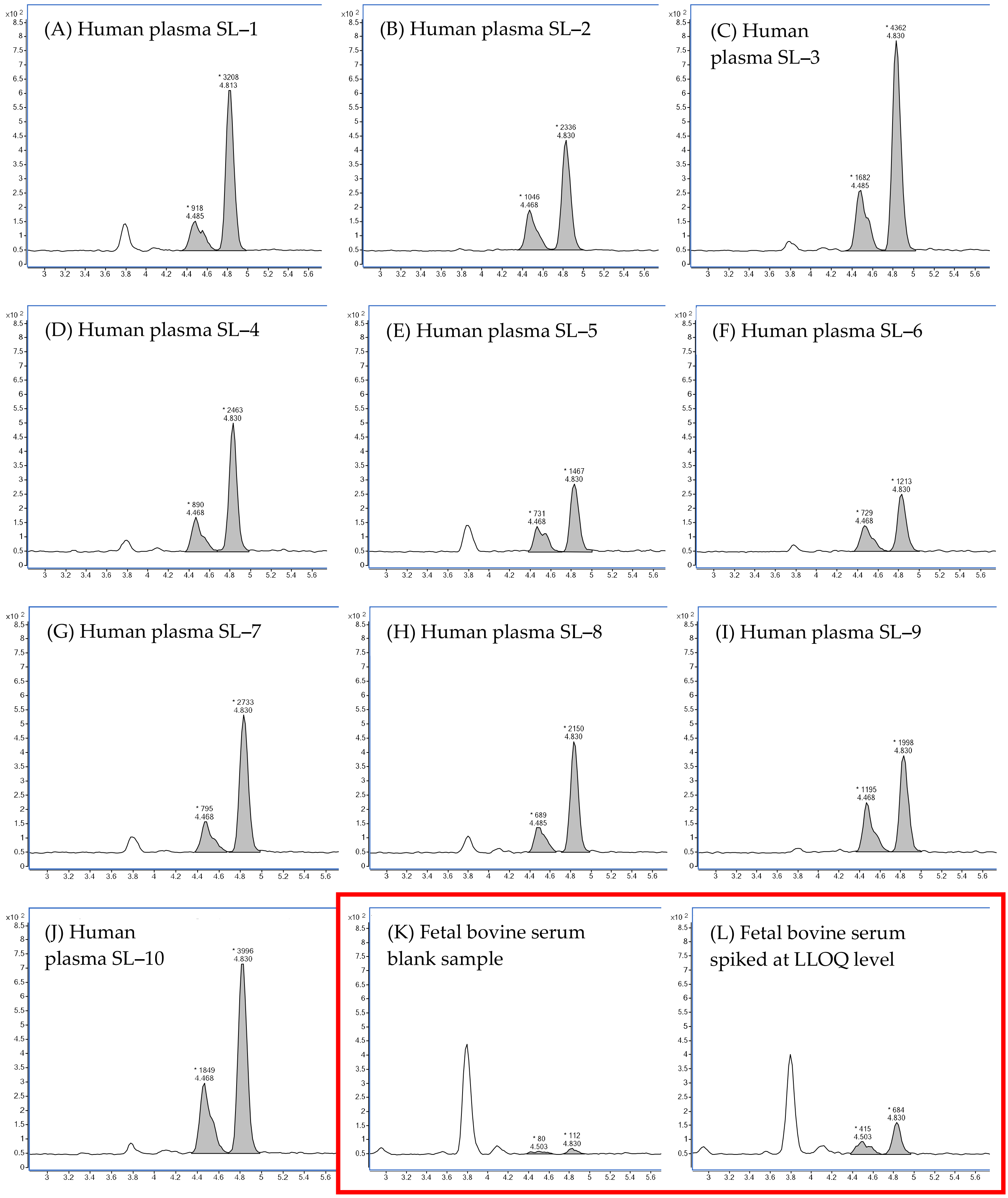

Figure S1 Extracted ion chromatograms of L–PFOS and br–PFOS in each biological matrix from selectivity test: (A–J) A total of 10 Thai blood donor's plasma samples; (K) FBS blank sample without endogenous interference; (L) FBS blank sample spiked at LLOQ level (L–PFOS=0.72 ng/mL, br–PFOS=0.19 ng/mL)

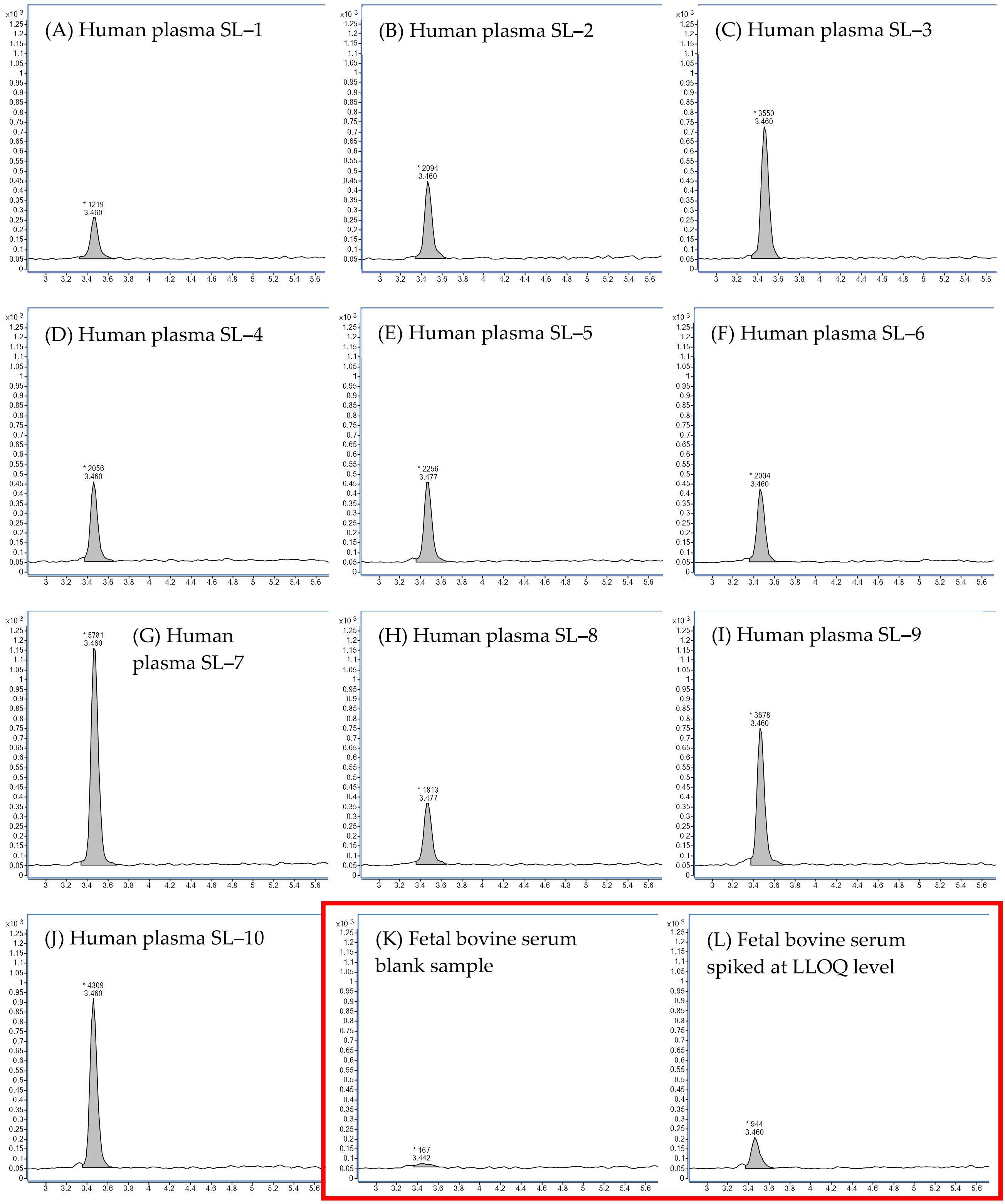

Figure S2 Extracted ion chromatograms of PFOA in each biological matrix from selectivity test: (A–J) A total of 10 Thai blood donor's plasma samples; (K) FBS blank sample without endogenous interference; (L) FBS blank sample spiked at LLOQ level (PFOA=0.49 ng/mL)

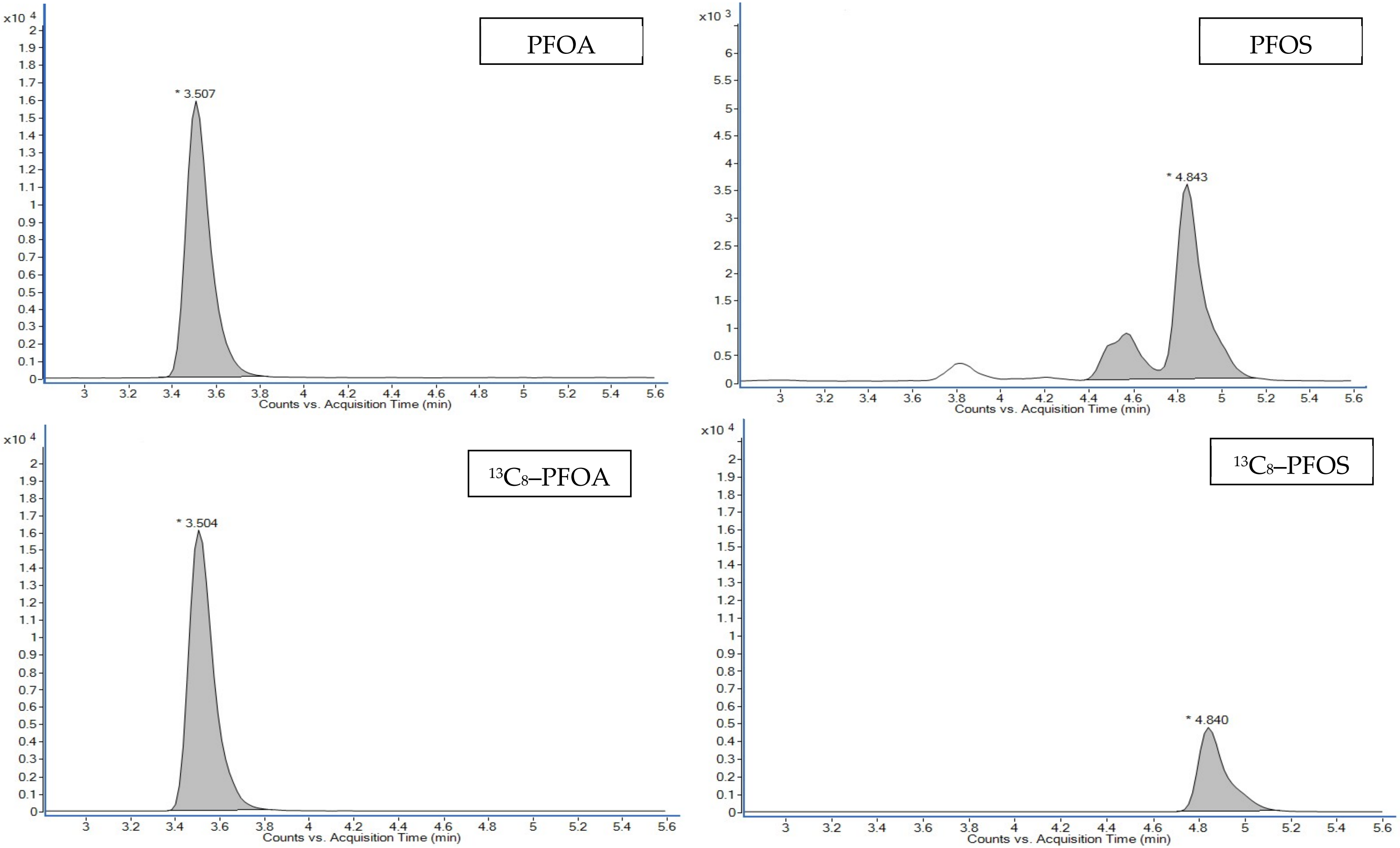

Figure S3 Extracted ion chromatograms of PFOA and PFOS standards and their isotopically labeled internal standards ($^{13}C_8$–PFOA and $^{13}C_8$–PFOS)
